# Perceived Changes of Attitudes and Behaviors of Seventh-Day Adventists During the COVID-19 Pandemic: Findings from a Cross-Sectional Survey in Germany

**DOI:** 10.1007/s10943-022-01580-y

**Published:** 2022-05-17

**Authors:** Arndt Büssing, Daniela Rodrigues Recchia, Lorethy Starck, Klaus van Treeck

**Affiliations:** 1 Professorship Quality of Life, Spirituality and Coping, Institute of Integrative Medicine, Faculty of Health, Witten/Herdecke University, 58313 Herdecke, Germany; 2 IUNCTUS - Competence Center for Christian Spirituality, Philosophical-Theological Academy, 48149 Münster, Germany; 3 Chair of Research Methods and Statistics in Psychology, Faculty of Health, Witten/Herdecke University, 58448 Witten, Germany; 4 Institute for Holistic Wellbeing, Resilience and Spirituality; affiliated institute at the Friedensau Adventist University, Bremen, Germany

**Keywords:** Perceived changes, COVID-19, Pandemic, Wellbeing, Fear of future, Awe, Spirituality, Seventh-Day-Adventists (SDA), Germany

## Abstract

To analyze which pandemic related changes of attitudes and behaviors were perceived by Seventh-day Adventists (SDA) and how these relate to wellbeing, a cross-sectional survey with standardized questionnaires was performed. Participants (*n* = 1,494) stated changes for *Relationships*, *Digital media usage* and *Nature/Silence/Contemplation*, but not for *Spirituality* or *Reflection of life*. Best predictors of psychological wellbeing (WHO-5) were Spiritual wellbeing, perceived Restrictions and Awe/Gratitude (*R*^2^ = .32). Mediation analyses (*R*^2^ = 0.51) revealed a mediation effect of Awe/Gratitude between spiritual to psychological wellbeing (*β* = 0.11, *p* < 0.0001). Perceived changes were less relevant to buffer the negative effects of the pandemic; instead, they were related to fears of future. More relevant to stabilize SDAs´ wellbeing was their spirituality.

## Introduction

During the COVID-19 pandemic many people were suffering from the outcomes of social restrictions due to the lockdowns (Rawson et al., 2020; Dubey et al., 2020). Studies have shown that the incidence of depressive states and anxiety disorders were increasing because of the pandemic (COVID-19 Mental Disorders Collaborators, 2021). Further, there was an increase in posttraumatic stress disorders, panic attacks and other psychic affections (Anjum et al., 2020). Some felt lonely, socially isolated and their psychological wellbeing was quite low (Passos et al., 2020; Büssing et al., 2020a, b, 2021a), while others reported that they had lost their (religious) faith during the pandemic (Büssing et al., 2022).

Nevertheless, people were not only experiencing loss and fears but also positive changes of attitudes and behaviors because of the pandemic (Büssing et al., 2020a, b). These changes refer to the dimensions *Nature/Silence/Contemplation* (i.e., perceiving nature more intensely, enjoying quiet times of reflection, paying more attention to what’s really important in life), *Spirituality* (i.e., praying/meditating more than before; being more interested in spiritual / religious issues), *Relationships* (more intense and more aware relationships with partners, family and friends), *Reflection on life* (i.e., being more concerned about the meaning and purpose of life and about the lifetime one has), and *Digital media usage* (to connect to friends and use inspiring websites) (Büssing et al., 2020b). Some of these changes could be interpreted in terms of “posttraumatic growth”, as people in response to difficult life situations may be more aware of their personal strengths, appreciate life, change priorities, focus on more intimate relationships, prosocial behaviors and spiritual development (Tedeschi & Calhoun, 1996; Tedeschi et al., 2018; Meichenbaum et al., 2006). These changes of attitudes and behaviors because of the pandemic could also be seen as a reappraisal coping, in the context of the transactional stress model (Lazarus & Folkman, 1984), where people try to adjust to the new situation of living in insecure times of the COVID-19 pandemic; this could be seen in terms of a “reappraisal of the person-environment relationship” (Lazarus, 1999). Thus, people intend to “revert” the pandemic related distress and regard it as a “challenge”, and thereby using their available resources (i.e., their faith) to cope (Lazarus & Folkman, 1984; Pargament, 1997).

During the first phase of the pandemic, these changes of attitudes and behaviors were perceived significantly stronger by older people, people with higher wellbeing, by those who can rely on their faith as a resource, and by those who perceived moments of wondering awe and gratitude (Büssing et al., 2020b). In the later phases of the pandemic, these perceptions were declining (with the exception of reflection of life), while peoples´ wellbeing decreased and the perception of stressors increased (Büssing et al., 2021a). During the later phases, moments of wondering awe and feelings of gratitude were one of the best predictors of these perceived positive changes in the pandemic (Büssing et al., 2021b). These feelings of wondering awe and gratitude are a perceptive aspect of spirituality that are experienced more often by older people and those who pray or meditate, but also by non-religious persons (Büssing, 2021). Such emotionally touching experiences may result in “feelings of interconnectedness, prosocial behavior, mindful awareness, and contribute to a persons´ meaning in life and wellbeing, and can also be a health relevant resource” (Büssing, 2021).

It is well known that a person´s faith or spirituality can be a buffer against adverse life events (Weber & Pargament, 2014), and thus it was assumed that this resource is relevant also to cope with the adverse effects of the corona pandemic (Kowalczyk et al., 2020; Pirutinsky et al., 2020; Asadzandi et al., 2020; Barmania & Reiss, 2020; Peteet, 2020; Koenig, 2020; Edara et al., 2021). Also for Muslims, it was stated that religion and religious coping may buffer health anxiety during the pandemic and may help to stabilize their wellbeing (Saud et al., 2021; Mahmood et al., 2021; Anchour et al., 2021). While these findings might be true for the first phases of the pandemic, it may not be true for all societies and specifically for the later phases of the pandemic (Büssing et al., 2022). In a larger German sample, it was shown that with the onset of the second lockdown the number of people who trusted in a “Higher Source” (God) decreased, while the proportion of people increased who stated that they have lost their faith because of the pandemic (Büssing et al., 2022). This was observed in younger and older age cohorts, and in Catholics, Protestants and also in people who did not state a religious affiliation but were nevertheless praying (Büssing et al., 2022). This would indicate some kind of “hope fatigue” as the “phases of insecurity and social isolation with lacking support by religious communities” were perceived as too long and are thus a challenge for the “religious coping capacities of religious/spiritual persons” (Büssing et al., 2022).

Therefore, we were interested how members of the Seventh-day Adventists (SDA) church coped with the pandemic. It is a rather small religious community in Germany (31,000 baptized members as compared to 20,000,000 Protestants) with high congregational cohesion among the parish members (ASTR, 2021). SDAs in general are known for their beneficial health behaviors (Orlich et al., 2013; Morton et al., 2017) and for their strict code of ethics and thus spiritual life (General Conference of Seventh-day Adventists, 2020). During the first lockdown SDA in Germany were well connected through digital media, and particularly the older members benefitted from this digital media application focusing on inspirational information contributing to their wellbeing (Büssing et al., 2021c). As one of the fundamental beliefs (#25) and hope of the SDAs is the “second coming of Christ” (General Conference of Seventh-day Adventists, 2020), the worldwide pandemic affecting millions of people could be interpreted by SDA as an indicator that this coming is near (Matthew 24, Luke 21, Mark 13). Therefore, one would expect that the pandemic would not have (negatively) affected SDA as strongly as compared to others religious people who do not share this belief as strongly, and that the pandemic is thus rather an ‘intensifier’ of their faith.

We thus aimed to analyze which changes of attitudes and behaviors related to the pandemic (in terms of social relations, perception of nature, silence and contemplation, intensifying their spirituality, reflection of life, and inspirational digital media usage) were observed in SDAs, and how these relate to their psychological and spiritual wellbeing. Further, we investigated perceptions of spiritual dryness, a specific form of religious struggle (Büssing et al., 2013, 2017), in response to the pandemic. In SDA´s, these phases were perceived significantly higher in women and in persons without a clear duty in the church (in contrast to men and persons in leading and other official roles) (Büssing et al., 2021d), and thus we specifically focused on gender and roles in the free church also in terms of perceived changes of attitudes and behaviors related to the pandemic. Further, we asked for SDAs´ hopeful intentions to pay more attention to each other (when the pandemic will once be overcome) and to contribute that the world will become fairer in the future on the one hand, and fearful perceptions that our society is falling apart more and more and to fear the future on the other hand. Both aspects, hope and fear, might be related to their expectation of Christ´s coming (General Conference of Seventh-day Adventists, 2020). Finally, we intended to clarify the impact of the perceived changes and indicators of spirituality on SDAs´ psychological wellbeing. As in a previous study, the perception of wondering awe and gratitude was identified as a relevant influencing variable with mediating effects on wellbeing (Büssing et al., 2021a), we performed mediation analyses with SDAs´ psychological wellbeing as outcome.

## Materials and Methods

### Participants

In this anonym cross-sectional survey, members of the German SDA were invited by emails to the regional coordinators, regional groups, Facebook groups, and SDA information journals to participate in an anonymous online survey with standardized measures. By filling in the anonymous questionnaire and by clicking the “Consent box” they consented to participate (“I have read the information provided and have understood the content of this information. I was able to make my decision unaffected. By anonymously completing and sending the questionnaire, I declare my consent to participate and to the anonymous further processing of the statements!”). Neither concrete identifying personal details nor IP addresses were recorded to guarantee anonymity. The study was reviewed and approved at the meeting of the presidents of the national SDA conferences in Dec 2021. The guidelines of the Helsinki Agreement and the voluntary nature of study participation have been confirmed.

Participants were recruited within two months from December 20, 2021, to February 22, 2022 (which represents the switch from the fourth to the fifth wave of the pandemic). Finally, 2,073 SDA were launching the questionnaire website, but not all consented to participate, or provided basic data. After elimination of these “non-responders” (*n* = 579), 1,494 participants remained in the sample who responded to the main relevant items addressing perceived changes (72% responder). Participants younger < 18 years were not processed. Participants were from different administrative units (conferences) of the SDA free church in Germany (32% Berlin/Mitteldeutschland, 26% Hanse, 17% Baden-Württemberg, 9% Bayern, 7% Nordrhein-Westfalen, 6% Mittelrheinisch); we included 4 participants from Austria and 7 from German language parts of Switzerland (0.8%).

### Measures

#### Perception of Changes

To assess which changes of attitudes and behaviors due to the Corona pandemic were observed by the participants, we used the 32-item *Perception of Change Questionnaire* (PCQ), which has good psychometric properties (Cronbach´s alpha = 0.91) (Büssing et al., 2020b). The instrument differentiates five main factors: 1) *Nature/Silence/Contemplation* (7 items, Cronbach´s alpha = 0.87; i.e., perceiving nature more intensely, enjoying quiet times of reflection, paying more attention to what’s really important in life); 2) *Spirituality* (5 items, Cronbach´s alpha = 0.83; i.e., praying / meditating more than before; being more interested in spiritual / religious issues; having confidence in a higher supporting power); 3) *Relationships* (6 items, Cronbach´s alpha = 0.80; (i.e., perceiving the relationship with partner / family more intensely; feeling closer to the people in my household; relationships have become important in which one can feel safe and at home); 4) *Reflection on life* (3 items, Cronbach´s alpha = 0.74; i.e., being more concerned about the meaning and purpose of life, being more concerned about the lifetime that I have; perceiving times of loneliness more); 5) *Digital media usage* (3 items, Cronbach´s alpha = 0.74; i.e., connected to friends via digital media; using more and more websites that inspire and stimulate) (Büssing et al., 2020b). All items were introduced by the phrase “Due to the current situation…”, which referred to the COVID-19 pandemic. The internal consistency of the PCQ in this sample is good (Cronbach´s alpha = 0.86).

A further, independent scale which is part of the PCQ addresses perceived *Restrictions* (c15 “I feel cut off from life”; c16 “I feel restricted in my freedom”; c17 “I lack social contacts”) with acceptable internal consistency (Cronbach´s alpha = 0.78) (Büssing et al., 2020b). Cronbach´s alpha in this sample is 0.75.

Additional items which are not integrated in the aforementioned factors address hopeful intentions (c25 “I have the hope that we (“afterwards”) as global mankind will pay more attention to each other and stick together”; c26 “I would like to work to ensure that the world becomes fairer in the future”) and also fearful perceptions (c27 “I realize that our society is falling apart more and more”; c28 “I rather fear the future “). These were used as informative single items.

Agreement or disagreement to all these items was scored on a 5-point scale (0—does not apply at all; 1—does not truly apply; 2—neither yes nor no; 3—applies quite a bit; 4—applies very much).

#### Psychological Wellbeing

To assess participants´ psychological well-being, we used the *WHO-Five Well-being Index* (WHO-5) (Bech et al., 2013). Representative items are “I have felt cheerful and in good spirits” or “My daily life has been filled with things that interest me”. Respondents assess how often they had the respective feelings within the last two weeks, ranging from “at no time” (0) to “all of the times” (5). Here, we report the sum scores ranging from 0 to 25. Scores < 13 would indicate reduced wellbeing in terms of even depressive mood. Cronbach´s alpha in this sample is 0.86.

#### Spiritual Wellbeing

In analogue to the psychological wellbeing as measured with the WHO-5, *Spiritual wellbeing* was addressed with 5 item that refer more closely to SDA´s spirituality (SpWb-5): “I felt close to God”; “I felt alive and fulfilled in my spiritual life”; “I felt like my prayers were really being answered”; “I was full of hope for the return of Jesus”; “I felt comforted by God in my challenges, worries and fears” (Büssing et al., 2021c). The respective feelings refer to the last two weeks and scored from “at no time” (0) to “all of the times” (5). Minimum scores are 0, and maximum scores are 25. Internal consistency of this SpWb-5 scale is good in this sample (Cronbach´s alpha = 0.88). In a study among SDA during the first lockdown, spiritual wellbeing correlated strongly with psychological wellbeing (WHO-5: *r* = 0.513) (Büssing et al., 2021c). In this study, the SWb-5 scale correlated moderately with wellbeing (WHO-5: *r* = 0.477), strongly with Awe/Gratitude (GrAw-7: *r* = 0.521) and strongly inversely with Spiritual Dryness (SDS-6: *r* = − 0.683).

#### Awe and Gratitude

Perceptions of wondering awe and subsequent feelings of gratitude is a perceptive aspect of spirituality which is also relevant to less or non-religious persons (Büssing et al., 2018). To address times of pausing for astonishment or “wonder” in specific situations (mainly in the nature), we measured perceived awe and subsequent feelings of gratitude with the 7-item *Awe/Gratitude* scale (GrAw-7) (Büssing et al., 2018). This scale has good psychometric properties (Cronbach’s alpha = 0.82) and uses items such as “I stop and then think of so many things for which I'm really grateful”, “I stop and am captivated by the beauty of nature”, “I pause and stay spellbound at the moment” and “In certain places, I become very quiet and devout”. Thus, Awe/Gratitude operationalized in this way is a matter of an emotional reaction towards an immediate and “captive” experience. All items were scored on a 4-point scale (0—never; 1—seldom; 2—often; 3—regularly), and finally transferred to a 100-point scale. Cronbach´s alpha in this sample is 0.86.

#### Spiritual Dryness

Perceptions of “spiritual dryness” were measured with the 6-item *Spiritual Dryness Scale* (SDS) which has good internal consistency (Cronbach’s alpha = 0.87) (Büssing et al., 2013). It addresses feelings that God is distant, that one’s prayers go unanswered, of being “spiritually empty” or not able to give any more (in terms of spiritual exhaustion) and, finally, feelings of being abandoned by God. We added a control item which asks for a “deep longing for God” (item SDS0). These items can be scored on a Likert scale ranging from not at all (1), rarely (2), occasionally (3), fairly often (4) to regularly (5). The SDS scores are mean scores and represent the perceived lack/shortage. Cronbach´s alpha in this sample is 0.88.

#### Frequency of Religious Practices and Perceptions

The frequency of spiritual/religious practices such as praying was assessed with a 4-grade scale ranging from “Less than once per week”, “Once per week”, “Several times per week” to “At least once per day” (Büssing et al., 2020b). SDA´s participation at worship meetings within the last 8 weeks in presence was categorized as “None”, “1–2 times”, “3–5 times” and “6–8 times”.

#### Corona Virus Infection and Vaccination

Participants were also asked whether they were already infected with the COVID-19 virus (“No, not to my knowledge”, “Yes, but without relevant symptoms”, “Yes, with distinct symptoms”, “Yes, with strong symptoms and treatment in hospital”), and whether they were vaccinated against the virus or not (“Already vaccinated”, “Considering vaccination”, “Rejection of vaccination”). These items were used as descriptive items and not as differentiating items.

### Statistical analyses

Descriptive statistics for demographic variables and for factors are presented as frequencies for categorical variables and as mean (± standard deviation, SD) for numerical variables. Between group comparisons for categorical variables was performed with Pearson’s Chi^2^ Independence Test. Analyses of variance (ANOVA), correlational (Spearman rho) and linear regression analyses (stepwise) were computed with SPSS 26.0. Mediation analysis was performed using SPSS 28.0.

Given the exploratory character of this study, we set a stricter significance level at p < 0.01. With respect to classifying the strength of the observed correlations, we adjusted the thresholds to r > 0.5 as a strong correlation, an r between 0.3 and 0.5 as a moderate correlation, an r between 0.2 and 0.3 as a weak correlation, and r < 0.2 as negligible or no correlation.

## Results

### Description of Study Participants

Among the participants (*n* = 1,494), gender is well balanced (50:50); their mean age is 53.4 ± 15.4, ranging from 18 to 94 years (Table 1). Within the sample, the majority has some duties in the local church (5% as pastors, and 20% with leading roles, 46% with other duties), while 28% have no circumscribed duties. Most were praying at a daily level (81%), and were often participating in worship meetings in physical presence (Table 1). At the time of recruitment, 70% were already vaccinated, 30% not; 19% stated that they were already infected with the COVID-19 virus (Table 1).

**Table 1:** Description of the sample (*n* = 1,494)

	*N*	%*	Mean ± SD [range]
*Gender*	1473	100	
Female	735	49.9	
Male	738	50.1	
No response	21		
*Age (years)*	1418	100	53.4 ± 15.4 [18–94]
< 40 years	321	22.6	
41–50 years	242	17.1	
51–60 years	361	25.5	
61–70 years	320	22.6	
> 70 years	174	12.3	
*Role in the free church*	1483	100	
Pastor	77	5.2	
Leading role in the local church	301	20.3	
Other duties in the local church	684	46.1	
Without duty in the local church	421	28.4	
*Frequency of private praying*	1490	100	
Less than once per week	33	2.2	
Once per week	51	3.4	
Several times per week	206	13.6	
At least once per day	1200	80.5	
*Participation at worship meetings within the last 8 weeks in presence*	1494	100	
None	305	20.4	
1–2 times	249	16.7	
3–5 times	273	18.3	
6–8 times	667	44.6	
*Corona vaccination*	1325	100	
Already vaccinated	927	70.0	
Considering vaccination	73	5.5	
Reject vaccination	325	24.5	
*COVID-19 infection*	1349	100	
No, not to my knowledge	1108	82.1	
Yes, but without relevant symptoms	137	10.2	
Yes, with distinct symptoms	95	7.0	
Yes, with strong symptoms treated in hospital	9	0.7	
*Hopeful intentions and fearful perceptions*			
c25 hope that we will pay more attention to each other and stick together	1482		2.4 ± 1.0 [0–4]
c26 work to ensure that the world becomes fairer in the future	1447		3.3 ± 0.8 [0–4]
c27 our society is falling apart more and more	1485		2.0 ± 1.2 [0–4]
c28 rather fear the future	1460		2.1 ± 1.0 [0–4]
*Indicators of Quality of life and Spirituality*			
Psychological wellbeing(WHO-5)	1399		14.5 ± 4.9 [5–25]
Spiritual wellbeing (SpWb-5)	1394		17.3 ± 5.4 ([5–25]
Awe/Gratitude (GrAw-7)	1398		62.6 ± 17.6 [0–100]
Spiritual Dryness (SDS-6)	1395		2.1 ± 0.8 [1–5]
*Longing for God (SDS0)*	1392	100	3.9 ± 1.0 [1–5]
Not at all /seldom	113	8.1	
Sometimes	285	20.5	
Often	559	40.2	
Regularly	435	31.3	
*Hope for the return of Jesus (SbWb item #4)*	1393	100	3.6 ± 1.5 [0–5]
At no time	0	0	
Some of the time	245	17.6	
Less than half of the time	93	6.7	
More than half of the time	153	11.0	
Most of the time	390	28.2	
All of the time	511	36.7	

^*^data refer to responders (100%)

Participants’ psychological wellbeing is in the lower “normal” range (34% with scores < 13, indicating depressive mood states), while their spiritual wellbeing is in the upper midrange (Table 1). Detailed analyses revealed that SDA without a duty in their church have significantly lower wellbeing scores (13.8 ± 5.2) as compared to persons with leading roles in the local church (15.1 ± 4.9) or pastors (14.8 ± 5.0) (F = 4.6, *p* = 0.003; ANOVA). Further, their spiritual wellbeing was significantly lower (16.4 ± 5.8) as compared to persons with leading roles in the church (18.2 ± 4.8) or pastors (17.1 ± 4.8) (*F* = 6.1, *p* < 0.0001; ANOVA). The effect size between the contrasting groups of SDA without a duty and with leading roles in the church are small for psychological wellbeing (Cohen’s *d* = 0.26) and small also for spiritual wellbeing (Cohen’s *d* = 0.33).

The perception of Awe/Gratitude (Table 1) is marginally lower than observed in a larger reference sample (Büssing, 2021). Phases of spiritual dryness were perceived by 14% often to regularly, by 35% sometimes, by 31% seldom and by 21% not at all. The SDS-6 mean score is in the lower midrange (Table 1).

Addressing their hopeful intentions (Table 1), 50% have “hope that we (“afterwards”) as global mankind will pay more attention to each other and stick together” (c25) and 36% are undecided, while 90%, stated that they “would like to work to ensure that the world becomes fairer in the future” (c26). In contrast, 38% perceive “that our society is falling apart more and more” (c27) and 27% are undecided; 33% “rather fear the future “ and 43% are undecided (Table 1). With respect to SDAs´ fundamental belief (#25) of the “second coming of Christ”, 37% were all of the time “full of hope for the return of Jesus” (SbWb item #4), and 28% most of the time (Table 1). Nevertheless, most have a deep longing for God (72% often / regularly, 21% sometimes, and 8% not at all / seldom) (Table 1).

### Perceived Changes of Attitudes and Behaviors due to the Pandemic

The strongest changes of attitudes and behaviors due to the pandemic were observed for *Digital media usage* and *Relationships*, followed by *Nature/Silence/Contemplation*, and the least strong changes were reported for *Spirituality*, *Reflection on life*, and *Restrictions* (Table 2). These changes were significantly stronger in women than men: They perceived *Nature / Silence / Contemplation* more intensely, adhered to their *Spirituality*, valued their *Relationships* and *Reflected on their life*; however, in trend also more women than men relied on *Digital media* to connect with others, while they all perceived the pandemic related *Restrictions* similarly (Table 2).

**Table 2:** Perceived changes in women and men, and persons with different roles in the free church

	Perceived changes related to the corona pandemic (PCQ)						
		Nature/Silence/Contemplation	Spirituality	Relationships	Reflection on life	Digital media usage	Restrictions
All (n = 1494)	Mean	56.38	51.43	61.91	51.47	67.15	51.66
	SD	17.55	14.00	18.36	19.10	20.87	27.29
*Gender*							
Women (n = 735	Mean	57.92	52.68	63.47	53.34	68.54	51.28
	SD	17.29	13.51	18.32	18.50	20.78	27.78
Men (n = 738)	Mean	54.74	50.22	60.33	49.66	65.98	52.33
	SD	17.66	14.38	18.30	19.49	20.71	26.75
F-value		12.21	11.45	10.85	13.83	5.63	0.54
p-value		< 0.0001	0.001	0.001	< 0.0001	.018	n.s
Age cohorts							
< = 40 years (n = 321)	Mean	52.42	50.86	61.92	52.17	65.14	57.33
	SD	17.90	13.83	16.64	18.63	20.52	28.01
41–50 years (n = 242)	Mean	55.62	51.12	61.64	48.83	65.07	53.20
	SD	18.68	13.60	18.27	19.04	21.53	25.31
51–60 years (n = 361)	Mean	56.43	51.10	61.16	49.87	65.67	52.01
	SD	16.44	13.34	18.20	19.11	20.69	27.22
61–70 years (n = 320)	Mean	58.79	51.61	62.52	52.57	68.89	48.48
	SD	15.91	14.19	18.46	18.24	20.73	27.17
> 70 years (n = 174)	Mean	60.38	53.63	63.91	56.23	73.73	44.92
	SD	18.13	15.39	19.89	19.88	19.64	27.33
F-value		8.23	1.31	0.76	4.94	6.81	7.52
p-value		< 0.0001	n.s	n.s	0.001	< 0.0001	< 0.0001
Role in the free church							
Pastor (n = 77)	Mean	52.03	50.19	58.60	48.70	65.04	51.41
	SD	16.71	14.31	20.03	19.87	18.93	22.76
Leading role in the local church (n = 301)	Mean	57.23	50.36	63.35	49.36	67.51	45.02
	SD	16.51	14.09	18.22	19.17	19.90	25.26
Other duties in the local church (n = 684)	Mean	56.29	51.67	62.24	51.75	67.51	52.20
	SD	17.49	13.87	17.96	18.62	20.13	28.17
Without duty in the local church (n = 421)	Mean	56.68	52.00	60.93	53.12	66.48	55.90
	SD	18.56	14.03	18.80	19.60	23.07	27.27
F-value		1.85	1.09	1.92	2.86	0.50	9.56
p-value		n.s	n.s	n.s	.036	n.s	< 0.0001

The age cohorts differ significantly for *Nature/Silence/Contemplation, Digital media usage* and *Restrictions* (which both are lowest in the younger ones and highest in the older ones), and for *Reflection of life* (which scored lowest in the “mid-agers” and highest in the older ones). *Spirituality* and *Relationships* did not differ within the age cohorts (Table 2).

With respect to their role in the free church, the different groups did not differ significantly for the positive changes (with the exception of a trend to more intensive *Reflections of life* in persons without a duty and lower scores in pastors), while they significantly differ in their perceptions of corona related *Restrictions*, which scored highest in SDA without a duty in their local church and lowest in persons with a leading role in their local church (Table 2).

### Correlations Between Perceived Changes and Indicators of Quality of Life and Spirituality

Most of the positively perceived changes were moderately interrelated (Table 3). However, *Digital media usage* was moderately related only to *Spirituality*, and marginally only with the other perceived changes (Table 3). The positive changes were further marginally only related to perceived *Restrictions*, both positively (*Spirituality* and *Reflection of life*) and negatively (*Nature / Silence / Contemplation* and *Relationships*).

**Table 3:** Correlations between perceived changes and indicators of wellbeing and spirituality, and hopeful intentions and fearful perceptions

	Perceived changes related to the corona pandemic (PCQ)					
	Nature/Silence/Contemplation	Spirituality	Relationships	Reflection on life	Digital media usage	Restrictions
Perceived changes						
Nature/Silence/Contemplation	1.000					
Spirituality	.439^**^	1.000				
Relationships	.472^**^	.319^**^	1.000			
Reflection on life	.394^**^	.440^**^	.296^**^	1.000		
Digital media usage	.189^**^	.382^**^	.162^**^	.187^**^	1.000	
Restrictions	− .107^**^	.192^**^	− .067^**^	.168^**^	− .013	1.000
Indicators of wellbeing and spirituality						
Wellbeing (WHO-5)	.226^**^	− .046	.113^**^	− .066	.045	− .387^**^
Spiritual wellbeing (SpWb-5)	.302^**^	.146^**^	.188^**^	.031	.106^**^	− .215^**^
Awe/Gratitude (GrAw-7)	.402^**^	.214^**^	.251^**^	.135^**^	.154^**^	− .214^**^
Spiritual Dryness (SDS-6)	− .205^**^	.034	− .104^**^	.074^**^	-.031	.274^**^
Hope for the return of Jesus (SbWb item #4)	.259^**^	.207^**^	.158^**^	.049	.130^**^	− .067
Deep longing for God (SDS0)	.207^**^	.230^**^	.117^**^	.119^**^	.102^**^	.062
Hopeful intentions and fearful perceptions						
c25 hope that we will pay more attention to each other and stick together	.221^**^	.189^**^	.225^**^	.305^**^	.157^**^	.001
c26 work to ensure that the world becomes fairer in the future	.031	.145^**^	.061	.000	− .045	.298^**^
c27 our society is falling apart more and more	− .090^**^	.183^**^	.027	.167^**^	− .043	.380^**^
c28 rather fear the future	.451^**^	.549^**^	.290^**^	.308^**^	.123^**^	.098^**^

^**^p < 0.01 (Spearman rho); moderate to strong correlations are highlighted (bold)

The factor *Nature / Silence / Contemplation* was the most relevant to be associated with indicators of wellbeing. It was moderately related to Awe/Gratitude and spiritual wellbeing, and weakly also with psychological wellbeing. Awe/Gratitude was further weakly related to *Spirituality* and *Relationships*. All other factors were either not significantly or marginally only related to these indicators of wellbeing (Table 3). In contrast, perceived *Restrictions* was negatively related to indicators of psychological and spiritual wellbeing and to Awe/Gratitude.

SDAs’ “hope for the return of Jesus” (SbWb item #4) and also their longing for God (SDS0) were weakly correlated with *Nature / Silence / Contemplation* and *Spirituality,* and marginally only with the other PCQ factors, but not significantly with *Restrictions* (Table 3).

The perception of Spiritual Dryness was weakly negative related to *Nature/Silence/Contemplation* and weakly positive to *Restrictions,* but not to changes in *Spirituality* or *Digital media usage* (Table 3).

### Correlations Between Perceived Changes and Hopeful Intentions and Fearful Perceptions

SDAs’ hope that we will “pay more attention to each other and stick together” was best and moderately associated with *Reflection of life*, and weakly also with *Nature/Silence/Contemplation* and *Relationships*, but not with perceived *Restrictions* (Table 3). In contrast, the intention to “work to ensure that the world becomes fairer in the future” when the pandemic is overcome was not relevantly associated with the perceived positive changes, but weakly with perceived *Restrictions.* However, this might also be due to the fact that there is not enough variance in their responses as most were agreeing anyway.

“Fear of the future” was strongly positively related to *Spiritualty*, and moderately positive to *Nature / Silence / Contemplation* and *Reflection of life*, and weakly positive also to *Relationships,* but not relevantly with the perceptions of *Restrictions* (Table 3). In contrast, the resigned statement that “our society is falling apart more and more” is not relevantly related to perceived positive changes, but moderately with *Restrictions*.

These hopeful intentions were only marginally related to SDAs’ “hope for the return of Jesus” or their deep longing for God, while their “fear of the future” was weakly positive associated with both variables and the perception that “our society is falling apart” marginally but inversely related (Table 4). Differentiating these associations in the respective “duty groups” revealed that in the small group of pastors, the hope for the “return of Jesus” was not significantly related with fear of future, and in persons with leading roles in the church only marginally positive (Table 4). In SDA with other duties in the church, both the hope and the longing for God were weakly associated with “fear of future”. Also in SDA without a duty hope for the “return of Jesus” was weakly associated with their “fear of future”, while this fear was moderately positive related to longing for God (Table 4). This “fear of the future” was strongly associated with more intense praying / meditation because of the pandemic (PCQ item c29) in both pastors (*r* = 0.651) and in the group of non-leading SDA (*r* = 0.655).

**Table 4:** Correlations between hopeful intentions and fearful perceptions and hope for the return of Jesus and longing for God in different cohorts

	All participants (*n* = 1393		Pastors (*n* = 73)		Leading role in the church (*n* = 280)		Other duties in the church (*n* = 648)		Without duty in the local church (*n* = 383)	
	Hope for the return of Jesus	Deep longing for God	Hope for the return of Jesus	Deep longing for God	Hope for the return of Jesus	Deep longing for God	Hope for the return of Jesus	Deep longing for God	Hope for the return of Jesus	Deep longing for God
Deep longing for God (SDS0)	.386^**^	1.000	.411**	1.000	.298**	1.000	.406**	1.000	.402**	1.000
c25 hope that we will pay more attention to each other and stick together	.108^**^	.116^**^	.345**	.215	.104	.047	.090	.107**	.097	.151**
c26 work to ensure that the world becomes fairer in the future	.159^**^	.122^**^	.066	.252	.148*	.006	.190**	.145**	.136**	.145**
c27 our society is falling apart more and more	− .186^**^	− .016	− .143	.022	− .195**	− .052	− .160**	− .017	− .223**	.006
c28 rather fear the future	.233^**^	.265^**^	.089	.209	.157**	.132	.266**	.285**	.244**	.314**

^**^p < 0.001 (Spearman rho); moderate to strong correlations are highlighted (bold)

### Predictors of SDA’s Psychological and Spiritual Wellbeing

To identify predictors of SDAs’ psychological wellbeing as dependent variable, we performed stepwise regression analyses (Table 5) that included items which were relevantly correlated with wellbeing. Thus, *Spirituality, Reflection of life, Digital media usage*, longing for God (SDS0), “hope that we will pay more attention to each other” (c25) and “fear of future” (c28) were not included in the model. Stepwise regression analyses revealed that spiritual wellbeing was the best predictor of psychological wellbeing as depending variable, explaining alone as much as 23% of variance (Table 5). Further relevant predictors are *Restrictions* (adding further 9% of explained variance) and Awe/Gratitude (adding further 3% of explaining variance). Further influences come from the perception that “our society is falling apart more and more” (c25), female gender, the intention to “work to ensure that the world becomes fairer in the future” (c26), and Spiritual Dryness. However, these four variables would add only 4% of further explained variance and are thus less relevant predictors. Without significant relevance in the regression model were age cohorts, *Nature/Silence/Contemplation* and *Relationships*.

**Table 5:** Predictors of wellbeing in SDA during the pandemic (stepwise regression analyses)

Dependent variable: psychological wellbeing(WHO-5) Model 7: *F* = 109.1, *p* < 0.0001; *R*^2^ = .38		Beta	*T*	*p*
	(constant)		10.270	< .0001
	Spiritual wellbeing (SpWb-5)	.242	7.121	< .0001
	Restrictions (PCQ)	− .203	− 8.033	< .0001
	Awe/Gratitude (GrAw-7)	.214	7.880	< .0001
	“Our society is falling apart more and more” (c27)	− .120	− 4.692	< .0001
	Female gender	.088	3.828	< .0001
	“Work to ensure that the world becomes fairer in the future” (c26)	− .090	− 3.758	< .0001
	Spiritual Dryness (SDS-6)	− .072	− 2.233	.026

Dependent variable: Spiritual Wellbeing (SpWb-5) Model 7: *F* = 315.8, *p* < 0.0001; *R*^2^ = .63		Beta	*T*	*p*
	(constant)		10.584	< .0001
	Spiritual Dryness (SDS-6)	− .498	− 24.485	< .0001
	Awe/Gratitude (GrAw-7)	.128	6.134	< .0001
	Deep longing for God (SDS0)	.164	8.960	< .0001
	psychological wellbeing(WHO-5)	.186	9.338	< .0001
	Frequency of private praying	.113	6.081	< .0001
	Spirituality (PCQ)	.093	5.222	< .0001
	Age cohorts	.057	3.227	.001

Spiritual wellbeing (SpWb-5) as dependent variable was negatively predicted by phases of Spiritual Dryness (explaining alone 49% of variance), and further positively by Awe/Gratitude (adding 6% of explained variance), longing for God (+ 3%) and psychological wellbeing (+ 2%). Additional influences on spiritual wellbeing came from frequency of praying, changes in *Spirituality,* and age, which together would explain only 2% additional variance and are thus less relevant. The other PCQ factors, *Restrictions* and also gender had no significant influence in this prediction model.

### Mediation Analyses

**Fig. 1:**
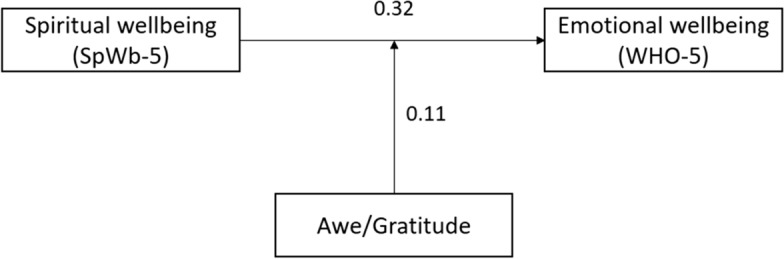
Mediation Analysis with Awe/Gratitude (GrAw-7) as mediator between Spiritual Well-Being (SpWb-5) and emotional Well-Being (WHO-5). The numbers indicate *β*-values with *p* < 0.0001. The total effect from the mediation analysis on well-being is estimated as *β* = 0.43 (*p* < 0.0001)

As Awe/ Gratitude was a relevant predictor for both wellbeing aspects, we investigated whether Awe/Gratitude may mediate the relationship between spiritual wellbeing and psychological wellbeing. In the following procedure of a mediation analysis, both the direct effect from spiritual wellbeing (SpWb-5) and Awe/Gratitude (GrAw-7) on psychological wellbeing (WHO-5) and also the mediation effect is evaluated. As shown in Fig. 1, spiritual wellbeing is a significant predictor for Awe/Gratitude (*β* = 1.70, *p* < 0.0001) and Awe/Gratitude is a relevant predictor for psychological wellbeing (*β* = 0.06, *p* < 0.0001). The mediation analysis explained 51% of the variability in the data (*R*^2^ = 0.51). The direct influence from spiritual well-being on psychological wellbeing was estimated as *β* = 0.32, *p* < 0.0001 and the mediation effect from Awe/Gratitude in the relationship between both wellbeing variables is statistically significant (*β* = 0.11, *p* < 0.0001). The total effect from the mediation analysis on well-being is estimated as *β* = 0.43, *p* < 0.0001.

## Discussion

Aim of this study was to analyze perceived changes of attitudes and behaviors of SDA within the pandemic. The timeframe of recruitment was between December 2021 and February 2022, which refers to the switch from the fourth wave (with the dominating Delta virus variant) to the fifth wave (with the dominating Omicron virus variant) in Germany. Compared to our study before the pandemic (Büssing et al., 2021d) and during the first lockdown (Büssing et al., 2021c), SDAs’ psychological wellbeing (WHO-5) scores were similar before the pandemic (14.8 ± 5.4) compared to the current wellbeing scores (14.5 ± 4.9), while they were surprisingly higher at the start of the pandemic (16.6 ± 5.1). Their spiritual wellbeing (SpWb-5) scores remained stable during the pandemic (17.5 ± 5.5 vs. 17.3 ± 5.4). Phases of spiritual dryness, a specific form of spiritual crisis, were perceived in a similar percentage as observed in SDA prior to the pandemic (Büssing et al., 2021d), and in similar proportions as in Catholic pastoral workers prior to the pandemic (Büssing et al., 2016, 2017). This means that the pandemic has not triggered more perceptions of spiritual dryness, and that God is not perceived as more “distant” and “non-responding” to prayers than before the pandemic.

### Changes of Attitudes and Behaviors Because of the Pandemic

Addressing perceived (positive) changes, SDA stated more intense relationships and more digital media usage to connect with others, but also more intense perceptions of nature and moments of silence and contemplation. No relevant changes were observed for their spirituality and reflection of life; restrictions due to the pandemic were less pronounced perceived. Compared to other cohorts in Germany, these positive changes were perceived much stronger in SDA. In the first phase of the pandemic, *Relationships* and *Nature / Silence / Contemplation* scored highest in a general population, followed by *Digital media usage* and *Reflection of life*, while *Spirituality* was not a relevant source (Büssing et al., 2021a). In the later phases of the pandemic (October 2020 to January 2021), these positive changes significantly decreased in those cohorts: *Relationships* and *Reflection of life* were still relevant, while *Digital media usage* (and *Nature/Silence/Contemplation* decreased in their relevance, and *Spirituality* lost its relevance (Büssing et al., 2021a). These decreases were not only observed in less or non-religious people, but also in religious people (Büssing et al., 2021a). The pattern of perceived changes is different in SDA, as they scored highest on *Digital media usage* and *Relationships*, followed by *Nature / Silence / Contemplation, Reflection on life* and *Spirituality*. This means, recruited in the later phases of the pandemic, SDA still perceive positive changes because of the pandemic. They might be more aware of these aspects in their life in terms of posttraumatic growth and reappraisal coping. This could be attributed either to a stricter religious faith conviction with high praying frequency and their habit to regularly participate in worship service even during the pandemic (see Table 1), but also to more digital support from the religious leaders (Büssing et al., 2021c)—or various other reasons.

Most of these changes in SDAs were perceived significantly stronger by women compared to men, and by older persons compared to younger ones. This pattern was found also in other cohorts (Büssing et al., 2020b). In a Danish sample prior to the pandemic, female SDA were further found to have a higher risk of being hospitalized for severe depression (Thygesen et al., 2013), indicating that they may be more sensitive to perception of burden. Nevertheless, SDAs’ role in the local community has no significant influence on the positively perceived changes, except for *Restrictions* which was perceived stronger by persons without a circumscribed duty in the church, but not significantly stronger by women. In line with previous findings addressing phases of spiritual dryness which were significantly more often perceived by SDA without a clear duty in their church (Büssing et al., 2021d), they are also more aware of pandemic related restrictions. It seems they perceived the negative sides of their spiritual and social life more intensively. In fact, detailed analyses revealed that SDA without a duty in their church have significantly lower psychological and spiritual wellbeing scores as compared to persons with leading roles in the local church or pastors; however, the effects sizes were small only.

Although digital media usage to connect with others and relationships were perceived as intensified, these perceptions were not relevantly related to SDA’s psychological and spiritual wellbeing or to perceptions of restrictions because of the pandemic (the associations are not significant at all or marginal only). However, more intense relations were weakly related to moments of wondering awe and gratitude, and marginally only for digital media usage. This means, these perceptions were reported by the participants, but these obviously had no deep impact on their wellbeing. However, the stronger the positive changes in *Relationships* were perceived, the stronger were SDAs’ hope that we will pay more attention to each other and stick together as a helping society when the pandemic is over (and vice versa). It might be that relationships are valued more intensely by the SDA as one may have experienced their fragility during the pandemic (with several people becoming infected, and some having complicated courses of the infection or were dying), and thus SDA may intend to protect that what is important to them – including the general social relationships as an ideal. However, being more aware for *Relationships* is thus not necessarily related to concrete (external) prosocial behaviors, and refers instead more to the closer (internal) circle of family and friends. In Catholics from Poland, relations in terms of social networks were reported to be larger in people with church attendance as compared to those without, which seems quite obvious, and a mediation effect of religious attendance”via social network size on loneliness and mental well-being” was verified (Okruszek et al., 2022). Interestingly, a small survey one year later revealed an increase of loneliness and decrease of wellbeing in both church attenders and non-attenders (Okruszek et al., 2022). Also in Germany, we observed a decrease of wellbeing with the duration of the pandemic in Catholics, Protestants and in praying people who stated no religious affiliation (Büssing et al., 2022). In this study among SDA from Germany, the frequency of participation of worship services in physical presence was only marginally related to psychological wellbeing (*r* = 0.15) or spiritual wellbeing (*r* = 0.19). The effects of religiosity (incl. participation in religious services and activities) on mental health are probably only small anyway, as shown in a meta-analysis by Garssen et al. (2021). Taken together, social relations are important to stabilize a person during the pandemic, but it is not necessarily the fact that one can attend religious services in physical presence (with several others) that would buffer the negative impact of social restrictions and would stabilize mental health. It is not the social network alone, but other factors that might be more important. Is it their spirituality?

SDAs’ perceptions regarding more aware aspects of *Spirituality* had no significant influence on their psychological wellbeing, and a marginal one only on their spiritual wellbeing and perceived *Restrictions*. Thus, these aspects of spirituality may be seen as a “habit” rather than a reactive form to cope which is intensified because of the pandemic. However, the factor *Spirituality* was strongly related to “fear of the future”, and this would indicate that intensifying this resource in response to the pandemic is in fact a reactive way to cope with the pandemic (related to fears and worries). This is all the more astonishing as the 1892 recommendation of Ellen G. White, cofounder of the SDA church, to meditate and contemplate the Bible on a daily basis (White, 2016) is regarded as still relevant (van Treeck, 2021). In fact, among the other perceived changes, *Spirituality* was best related to *Reflection of life*, which implies rather “worrying” aspects (such as being more concerned about the meaning and purpose of life and about the lifetime one has have, but also more intense perceptions of loneliness). This would underline that intensifying one´s spirituality during the pandemic is a strategy to cope with fears and worries, while it is nevertheless not necessarily contributing to psychological wellbeing. This might be different in other religious groups as shown for Muslims (Saud et al., 2021; Mahmood et al., 2021; Anchour et al., 2021).

The source with the most relevant positive associations was *Nature/Silence/Contemplation*. It was moderately related to perceptions of wondering awe and gratitude, to spiritual wellbeing, weakly to psychological wellbeing and to the hope that mankind will stand together–and moderately also with “fear of the future”. While the aforementioned associations are plausible because being aware of God´s creation and enjoying quiet times of contemplation (and praying) may contribute to wellbeing, it is nevertheless also a chance to reflect on what is important in life, and to reflect on the time one has in this lifetime (referring to the moderate positive associations with *Relationships* and *Reflection of life*). During these quiet times in silence and prayer one may also become more aware of one’s own fears and worries, and could bring these fears before God. This would also explain the weak association between *Nature/Silence/Contemplation* and SDAs´ “hope for the return of Jesus” and their longing for God. Women and older persons seem to be more aware of this resource for coping with the pandemic.

### Hopeful Intentions and Fear of Future

It was interesting to see that SDAs’ “hope for the return of Jesus” and their longing for God was expressed quite high. Both are essential parts of SDA fundamental beliefs (General Conference of Seventh-day Adventists, 2020). However, this does not mean that both, the hope and the longing, are relevantly related to their intentions that the world will become fairer when the pandemic is over or that we all will pay more attention to each other and stick together as mankind. These associations are marginal only. Instead, both the expectation and the longing, which are moderately interconnected, are positively and at least weakly related to SDAs’ “fear of the future”. For SDA that hope should be a living concept and thus one would expect a negative association: The expectation could be a matter of joy as an eschatological perspective and not fear. However, because of its negative association it would again indicate that the “hope for the return of Jesus” might be rather a theoretical concept related to fear. One may consider that Jesus’ second coming is said to be a matter of Final Judgement where some are approved and others condemned (Matthew 25:31–46), and thus this belief can be related to fear when one assumes that the own merits are not enough to be approved. In contrast, the perception that our society is “falling apart” is negatively related to both variables (hope for Jesus’ coming and longing for God), indicating that strong longing and hope are associated with less “end time experience” (in terms that the society is falling apart). Thus, both indicators of SDAs’ spirituality (hope and longing) are buffering the resigned perception on the one hand, but not buffering their fear of future on the other hand. One may fear the concrete future, but may have hope as a spiritual perspective. In pastors and SDA with leading roles in their church the association between both the hope of the return of Jesus and longing for God, were not relevantly associated with fear of the future, while this association was weakly to moderately positive in SDA with other or no duties in the church (representing the “normal” congregation members). For leaders in the church the core beliefs are thus not related to fear for future, probably because they are stronger in their faith convictions or have a profound theological interpretation at hand, while for the other community members one may assume that their longing for God (as a helping resource) is meant to compensate their (worldly) fears and worries. This assumption would be supported by the finding that more intense praying / meditation because of the pandemic was strongly related to “fear of the future”, both in persons with leading duties and also in pastors, indicating that they all bring their fears before God during their prayers / meditation.

### Wellbeing and Awe Perceptions

One of the further aims of this study was to analyze which of the aforementioned variables may have a significant influence on SDAs’ wellbeing during the pandemic. Regression analyses indicated that psychological wellbeing was predicted best by spiritual wellbeing and by Awe/Gratitude, with a further relevant negative effect of perceived *Restrictions*. It is plausible that the perception of being restricted in social life and that “our society is falling apart more and more” will have a negative impact on wellbeing. Yet, it is worth mentioning that the intention to work “towards the world becoming fairer in the future” is a negative predictor of wellbeing, probably because it is the reaction of the frustration experience of the pandemic related restrictions and that our society is becoming more and more divided.

SDAs’ spiritual wellbeing was predicted best by less frequent experiences of Spiritual dryness, which is a specific form of spiritual crisis (Büssing & Dienberg, 2019, 2021; Büssing et al., 2021a), and positively by their ability to stop in wondering awe with subsequent feelings of gratitude, by longing for God, frequency of private (spontaneous) praying, and psychological wellbeing. SDAs´ spiritual wellbeing is thus a matter of being connected with God, who is perceived as close and responding to prayers, and as accessible in different situations, either in nature or in prayers.

As a crucial finding was the prominent influence of Awe/Gratitude for both wellbeing aspects, and because Awe/Gratitude was identified in earlier studies as a potential mediator of psychological wellbeing (Büssing et al., 2021b), its mediating role was also analyzed in this study. We found a direct influence from spiritual well-being on psychological well-being, and a mediation effect from Awe/Gratitude in the relationship between both well-being variables. This resource implies an ability to mindfully stop in wondering awe with perceptions of gratitude. It means to be aware of God’s creation and of being emotionally and spiritually touched in different situations and encounters—even in difficult situations (Büssing, 2021). This ability was found to be more pronounced in women, older people and praying / meditating persons as compared to men, younger ones, and non-religious people (Büssing et al., 2018; Büssing, 2021). Yet, this resource does not buffer the negative effects of the pandemic–but helps to focus on the still positive aspects of life (Büssing et al., 2021b).

To what extent the celebration of the Sabbath as a regular weekly day of rest (fundamental belief #20) may contribute to the described findings, remains unclear. Adventists are advised to spend a whole day (24 h Sabbath from sunset Friday to sunset Saturday) with rest, leisure, recreation, worship and fellowship, family, and in nature. Perhaps these quiet and reflexive times contribute to integrate the described experiences more easily into everyday life. Further studies could shed light on this. If this kind of Sabbath observance has such an influence on Awe/Gratitude, then this experience could be an impulse for observing a strict weekly day of rest in other religious or non-religious contexts, too. However, in our study before the pandemic (Büssing et al., 2021d), strictness of Sabbath keeping was marginally only related to SDAs’ perception of Awe/Gratitude (*r* = 0.14), while their frequency of private (spontaneous) prayers was moderately related (*r* = 0.31). Spontaneous prayers are thus more relevant as this would indicate a direct response towards ‘touching’ experiences (i.e., to give thanks, praise, but also to articulate fears and worries). While the frequency of spontaneous praying is not relevantly contributing to psychological wellbeing (*r* = 0.10), it is moderately related to SDAs´ spiritual wellbeing (*r* = 0.35) and thus a relevant resource.

## Limitations

This is a cross sectional study and thus no causal interpretations can be drawn. In some cases, it is plausible to assume directions of influences, but these can be verified only in longitudinal studies. For this study, we have reference data from SDA from the start of the pandemic only for some variables (Büssing et al., 2021c). These data would indicate that their wellbeing was in fact decreasing, as it was observed in an other cohorts from Germany, too (Büssing et al., 2021a, 2022).

With respect to representatives of our sample, 59% of the SDA in Germany are women (mean age 57.8 years) and 41% men (mean age 55.5 years). In our current analysis, 50% women and 50% men have participated. Their mean ages are 51.8 ± 14.9 (women) and 54.9 ± 15.7 (men). Thus, in our sample 9% less women than one could expect have participated, and also the mean age was slightly lower as compared to the general group of SDA. Despite these differences, our sample fits to the expected age ranges.

As this is an online study, SDA with no access to the internet cannot participate. However, as the SDA have very early started to establish digital media usage (i.e., text messages, email, messenger services, satellite evangelism and mission, service broadcasts, and video conferencing), also several of the elderly are digitally interconnected.

## Conclusions

Similar to all other religious persons, SDA had to cope with the outcomes of the pandemic. Apart from perceived social restrictions and fears and worries because of the pandemic, their wellbeing scores were higher at the start of the pandemic, and their spiritual wellbeing remained stable. While other cohorts in Germany stated a loss of faith because of the pandemic (Büssing et al., 2022), it seems that SDA have found more stability in their faith and in their community. They noticed positive changes of attitudes and behaviors because of the pandemic, yet the higher awareness of the underlying resources will not necessarily buffer the pandemic related restrictions or their fears and worries. Instead, these changes made them more aware to value life in its complexity more than before, and probably have sensitized their experience of the fragility of human life. The experience of the Sacred in nature and in reflective times of silence (before God) were related to their spiritual and psychological wellbeing and to their ability to stop in wondering awe (with subsequent feelings of gratefulness)—despite of the pandemic. The outstanding role of Awe/Gratitude was underlined as it was a relevant predictor of both psychological and spiritual wellbeing, and as a mediator of the positive link between spiritual to emotional wellbeing. Thus, this resource should be fostered, as it was experienced also by less or non-religious persons (Büssing et al., 2021b, Büssing, 2021) and could help to perceive the still positive aspects in life, even during the pandemic. A stabilizing source would thus be awareness of one’s relation to God that may find its expression in times of silence, in nature and prayer. Yet, this requires some mental stability. In fact, the faith inherent struggles and the fears of the final Judgement can be a burden for religious people. Thus, church leaders have to carefully respond to these struggles which are often not communicated by community members, as these are assumed to be a matter of “shame” or “spiritual weakness” (Büssing & Dienberg, 2019). As it was evident that particularly SDA without a duty in their community perceive the social restrictions of the pandemic, and as they more often experience phases of spiritual dryness (Büssing et al., 2021d), this group should be considered as more vulnerable than those who are better integrated with clear duties (and thus recognition by others) in the local church.

## Data Availability

According to the data protection regulations, the data set cannot be made publicly available. Data are however available from the authors upon reasonable request.

## References

[CR1] Achour M, Souici D, Bensaid B, Zaki NBA, Alnahari AAA (2021). Coping with anxiety during the COVID-19 pandemic: A case study of academics in the muslim world. Journal of Religion and Health.

[CR2] Anjum S, Ullah R, Rana MS, Khan HA, Memon FS, Ahmed Y, Jabeen S, Faryal R (2020). COVID-19 pandemic: A serious threat for public mental health globally. Psychiatria Danubia.

[CR3] Asadzandi M, Abolghasemi H, Javadi M, Sarhangi F (2020). A Comparative assessment of the spiritual health behaviors of the Iranian muslim in the COVID-19 pandemic with religious evidence. Journal of Military Medicine.

[CR4] ASTR - Office of Archives, Statistics and Research (2021). 2021 Annual Statistical Report Vol. 3. *Report of the General Conference of Seventh-day Adventists’ 2020 Statistics*. Seventh-day Adventist Church, Available online: https://documents.adventistarchives.org/Statistics/ASR/ASR2021A.pdf?_ga=2.249613248.71027430.1646231106-973589172.1646231106&_gl=1*1sw0kzt*_ga*OTczNTg5MTcyLjE2NDYyMzExMDY.*_ga_2VBYH6KEBQ*MTY0NjIzMTEwNS4xLjEuMTY0NjIzMTIxNS4w

[CR5] Barmania S, Reiss MJ (2020). Health promotion perspectives on the COVID-19 pandemic: The importance of religion. Global Health Promotion.

[CR6] Bech P, Olsen LR, Kjoller M, Rasmussen NK (2013). Measuring well-being rather than the absence of distress symptoms: A comparison of the SF-36 mental health subscale and the WHO-Five well-being scale. International Journal of Methods in Psychiatric Research.

[CR7] Büssing, A., & Dienberg, T. (2021). Gottes Unverfügbarkeit und die dunkle Nacht. Vom Umgang mit der geistlichen Trockenheit [God's Unavailability and the Dark Night. Dealing with spiritual dryness]. Regensburg: Pustet Verlag.

[CR8] Büssing A (2021). Wondering Awe as a perceptive aspect of spirituality and its relation to indicators of wellbeing: Frequency of perception and underlying triggers. Frontiers of Psychiatry.

[CR9] Büssing A, Baumann K, Jacobs C, Frick E (2017). Spiritual dryness in catholic priests: Internal resources as possible buffers. Psychology of Religion and Spirituality.

[CR10] Büssing A, Baumann K, Surzykiewicz J (2022). Loss of faith and decrease of trust in Higher Source during the corona pandemic. Journal of Religion and Health.

[CR11] Büssing A, Dienberg T (2019). Geistliche Trockenheit - empirisch, theologisch, in der Begleitung.

[CR12] Büssing A, Frick E, Jacobs C, Baumann K (2016). Spiritual dryness in non-ordained catholic pastoral workers. Religions.

[CR13] Büssing A, Günther A, Baumann K, Frick E, Jacobs C (2013). Spiritual dryness as a measure of a specific spiritual crisis in Catholic priests: Associations with symptoms of burnout and distress. Evidence-Based Complementary and Alternative Medicine.

[CR14] Büssing A, Hübner J, Walter S, Gießler W, Büntzel J (2020). Tumor patients´ perceived changes of specific attitudes, perceptions and behaviors due to the Corona pandemic and its relation to reduced wellbeing. Frontiers in Psychiatry.

[CR15] Büssing A, Recchia D, Baumann K (2018). Validation of the gratitude/awe questionnaire and its association with disposition of gratefulness. Religions.

[CR16] Büssing A, Recchia DR, Dienberg T, Surzykiewicz J, Baumann K (2021). Dynamics of perceived positive changes and indicators of wellbeing within different phases of the COVID-19 pandemic. Frontiers in Psychiatry.

[CR17] Büssing A, Recchia DR, Dienberg T, Surzykiewicz J, Baumann K (2021). Awe/Gratitude as an experiential aspect of spirituality and its association to perceived positive changes during the COVID-19 pandemic. Frontiers in Psychiatry.

[CR18] Büssing A, Recchia DR, Hein R, Dienberg T (2020). Perceived changes of specific attitudes, perceptions and behaviors during the Corona pandemic and their relation to wellbeing. Health and Quality of Life Outcomes.

[CR19] Büssing A, Starck L, van Treeck K (2021). Experience of spiritual dryness and acedia symptoms in Seventh-day Adventists. Journal of Religion and Health.

[CR20] Büssing A, Starck L, van Treeck K (2021). Wellbeing and digital media usage to strengthen the faith of Seventh-Day Adventists during the Corona pandemic. Journal of Applied Christian Leadership.

[CR21] COVID-19 Mental Disorders Collaborators (2021). Global prevalence and burden of depressive and anxiety disorders in 204 countries and territories in 2020 due to the COVID-19 pandemic. Lancet.

[CR22] Dubey S, Biswas P, Ghosh R, Chatterjee S, Dubey MJ, Chatterjee S, Lahiri D, Lavie CJ (2020). Psychosocial impact of COVID-19. Diabetes & Metabolic Syndrome: Clinical Research & Reviews.

[CR23] Edara IR, Del Castillo F, Ching GS, Del Castillo CD (2021). Religiosity, emotions, resilience, and wellness during the COVID-19 pandemic: A study of Taiwanese university students. International Journal of Environmental Research and Public Health.

[CR24] Garssen B, Visser A, Pool G (2021). Does spirituality or religion positively affect mental health? Meta-analysis of longitudinal studies. The International Journal for the Psychology of Religion.

[CR25] General Conference of Seventh-day Adventists: *28 Fundamental Beliefs.* https://www.adventist.org/wp-content/uploads/2020/06/ADV-28Beliefs2020.pdf Last access: February 298, 2022.

[CR26] Koenig HG (2020). Maintaining health and well-being by putting faith into action during the COVID-19 pandemic. Journal of Religion and Health.

[CR27] Kowalczyk O, Roszkowski K, Montane X, Pawliszak W, Tylkowski B, Bajek A (2020). Religion and faith perception in a pandemic of COVID-19. Journal of Religion and Health.

[CR28] Lazarus RS (1999). Stress and Emotion.

[CR29] Lazarus RS, Folkman S (1984). Stress, Appraisal, and Coping.

[CR30] Mahmood QK, Jafree SR, Sohail MM, Akram MB (2021). A cross-sectional survey of Pakistani muslims coping with health anxiety through religiosity during the COVID-19 pandemic. Journal of Religion and Health.

[CR31] Meichenbaum D, Calhoun LG, Tedeschi RG (2006). Handbook of posttraumatic growth: Research and practice.

[CR32] Morton KR, Lee JW, Martin LR (2017). Pathways from religion to health: Mediation by psychosocial and lifestyle mechanisms. Psychology of Religion and Spirituality.

[CR33] Okruszek Ł, Piejka A, Żurek K (2022). Take me to (the empty) church? Social networks, loneliness and religious attendance in young polish adults during the COVID-19 pandemic. Journal of Religion and Health.

[CR34] Orlich MJ, Singh PN, Sabate J, Jaceldo-Siegl K, Fan J, Knutsen S, Beeson WL, Fraser GE (2013). Vegetarian dietary patterns and mortality in adventist health study 2. JAMA Internal Medicine.

[CR35] Pargament KI (1997). The Psychology of Religion and Coping: Theory, Research.

[CR36] Passos L, Prazeres F, Teixeira A, Martins C (2020). Impact on mental health due to COVID-19 Pandemic: Cross-sectional study in Portugal and Brazil. International Journal of Environmental Research and Public Health.

[CR37] Peteet JR (2020). COVID-19 anxiety. Journal of Religion and Health.

[CR38] Pirutinsky S, Cherniak AD, Rosmarin DH (2020). COVID-19, mental health, and religious coping among American orthodox jews. Journal of Religion and Health.

[CR39] Rawson T, Brewer T, Veltcheva D, Huntingford C, Bonsall MB (2020). How and when to end the COVID-19 lockdown: An Optimization approach. Frontiers in Public Health.

[CR40] Saud M, Ashfaq A, Abbas A, Ariadi S, Mahmood QK (2021). Social support through religion and psychological well-being: COVID-19 and coping strategies in Indonesia. Journal of Religion and Health.

[CR41] Tedeschi RG, Calhoun LG (1996). The posttraumatic growth inventory: Measuring the positive legacy of trauma. Journal of Traumatic Stress.

[CR42] Tedeschi RG, Shakespeare-Finch J, Taku K, Calhoun LG (2018). Posttraumatic Growth: Theory, Research, and Applications.

[CR43] Thygesen LC, Dalton SO, Johansen C, Ross L, Kessing LV, Hvidt NC (2013). Psychiatric disease incidence among danish seventh-day adventists and baptists. Social Psychiatry and Psychiatric Epidemiology.

[CR44] van Treeck, K. (2021). Spirituelle Krisen im Leben und Schrifttum der Mystikerin Ellen Gould White [Spiritual Crises in the Life and Writings of the Mystic Ellen Gould White]. In: Büssing, A., & Dienberg, T. (eds). Gottes Unverfügbarkeit und die Dunkle Nacht. Vom Umgang mit der geistlichen Trockenheit [God's Unavailability and the Dark Night. Dealing with spiritual dryness]. Aschendorf Verlag Regensburg, pp 35–53.

[CR45] Weber SR, Pargament KI (2014). The role of religion and spirituality in mental health. Current Opinion in Psychiatry.

[CR46] White EG (2016). Steps to Christ.

